# Dynamic Urinary Albumin/Creatinine Ratio Patterns Predict Adverse Outcomes in HFpEF: TOPCAT Cohort Analysis

**DOI:** 10.1002/clc.70446

**Published:** 2026-08-14

**Authors:** Weiping Xiao, Zhiyan Wang, Di‐en Yan, Min‐an Peng, Xinyuan Zhou

**Affiliations:** ^1^ Department of Endocrinology Ji'an Central People's Hospital Ji'an Jiangxi China; ^2^ Beijing Anzhen Hospital Capital Medical University Beijing China; ^3^ Department of Cardiology Ji'an Central People's Hospital Ji'an Jiangxi China

**Keywords:** heart failure with preserved ejection fraction (HFpEF), risk prediction, trajectory analysis, urinary albumin‐to‐creatinine ratio

## Abstract

**Background:**

Urinary albumin‐to‐creatinine ratio (uACR) is a recognized cardiovascular risk marker, but its dynamic changes in heart failure with preserved ejection fraction (HFpEF) remain underexplored. We aimed to identify distinct uACR trajectory patterns and their associations with clinical outcomes in HFpEF patients.

**Methods:**

We analyzed 746 HFpEF patients with ≥ 3 uACR measurements from the TOPCAT trial. Latent Class Trajectory Modeling identified uACR patterns. The primary outcome was cardiovascular death, aborted cardiac arrest, or heart failure hospitalization. Cox proportional hazards models assessed associations between trajectories and outcomes.

**Results:**

Three uACR trajectory patterns emerged: stable (70.5%), decreasing (16.5%), and fluctuating high (13.0%). The primary endpoint occurred in 15.02%, 25.20%, and 36.08% of patients, respectively (*p* < 0.001). In adjusted models, the fluctuating high group showed increased risk for the composite endpoint (HR 1.91, 95% CI 1.25–2.91) and heart failure hospitalization (HR 1.91, 95% CI 1.19–3.06), while the decreasing group showed an association with myocardial infarction based on few events (HR 3.00, 95% CI 1.33–6.76). Adding trajectory information produced a modest improvement in risk prediction (C‐index 0.758 to 0.765; AUC 0.786 to 0.792 at 4 years and 0.832 to 0.845 at 6 years, *p* = 0.0149).

**Conclusion:**

This study first identified distinct uACR trajectory patterns in HFpEF patients. The fluctuating high trajectory was associated with increased cardiovascular risk and heart failure hospitalization, while the association between the decreasing trajectory and myocardial infarction was exploratory and requires validation.

## Graphical abstract

Among 746 HFpEF patients in TOPCAT, latent class modeling identified three uACR trajectories: stable, decreasing, and fluctuating high. The fluctuating high trajectory independently predicted the composite of cardiovascular death, aborted cardiac arrest, or heart failure hospitalization, and adding trajectory data modestly improved risk prediction.

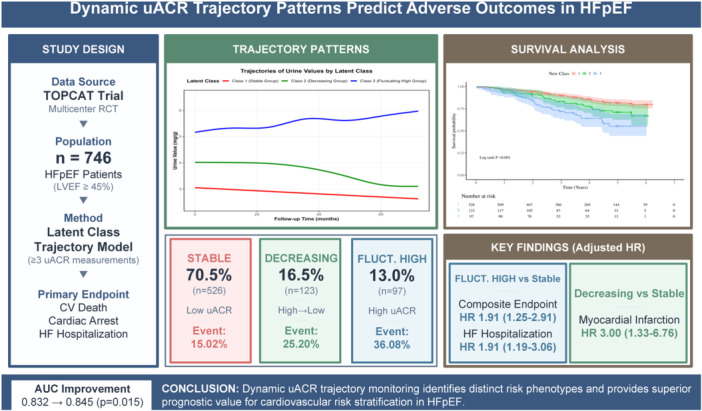

## 1 Introduction

Heart failure with preserved ejection fraction (HFpEF) refers to a condition where left ventricular ejection fraction (LVEF) is normal or near normal (typically ≥ 50%), yet patients exhibit typical signs and symptoms of heart failure. The main characteristics of HFpEF include diastolic dysfunction, reduced cardiac compliance, and frequent comorbidities such as hypertension, obesity, diabetes, and age‐related changes [1]. Epidemiological data indicate that HFpEF has become the predominant type of heart failure globally, accounting for approximately 50% of all heart failure cases, with its prevalence continuing to rise due to population aging and increasing metabolic diseases [2]. Despite similar mortality rates to heart failure with reduced ejection fraction (HFrEF), HFpEF presents distinct challenges with its higher burden of comorbidities, increased hospitalization rates, and significantly impaired quality of life [3]. Treatment options for HFpEF remain limited, with no therapies definitively proven to reduce mortality. Current management primarily relies on diuretics, mineralocorticoid receptor antagonists, and lifestyle modifications [4]. The heterogeneous pathophysiology and diverse clinical phenotypes of HFpEF complicate both diagnosis and treatment strategies, underscoring the critical need for improved risk stratification tools and personalized therapeutic approaches [5].

Microalbuminuria is typically defined as urinary albumin excretion of 30–300 mg/24 h or urinary albumin‐to‐creatinine ratio (uACR) of 30–300 mg/g (3.4–34 mg/mmol), and is widely recognized as a sensitive marker of glomerular filtration membrane damage and systemic endothelial dysfunction [6]. In clinical practice, uACR measurement is simple and convenient, requiring only a random urine sample, making it a practical tool for screening early kidney damage and assessing cardiovascular risk [7]. From a pathophysiological perspective, microalbuminuria reflects systemic endothelial dysfunction, low‐grade inflammation, and increased oxidative stress, mechanisms that also contribute to the development and progression of cardiovascular disease [8]. Substantial epidemiological evidence confirms that microalbuminuria is significantly associated with cardiovascular events and all‐cause mortality. In the general population, microalbuminuria increases cardiovascular mortality risk by 57%, cardiovascular event risk by 69%, and all‐cause mortality risk by 65% [9]. Long‐term follow‐up cohorts such as the Hoorn Study have found that microalbuminuria is associated with a 2–4‐fold increase in cardiovascular mortality risk, remaining an independent predictor after multivariate adjustment [10]. In specific high‐risk populations, the prognostic value of microalbuminuria is even more significant. For diabetic patients, the HOPE study demonstrated that microalbuminuria is associated with a 1.83‐fold increase in major cardiovascular event risk [11]. In hypertensive or borderline hypertensive patients, microalbuminuria is the strongest predictor of ischemic heart disease, with an adjusted cardiovascular event risk increase of approximately 3.5‐fold [12]. For patients with established heart failure, regardless of ejection fraction, increased urinary albumin excretion is independently associated with increased mortality and rehospitalization rates [13, 14].

In recent years, researchers have increasingly focused on the impact of dynamic changes in uACR on prognosis. Compared to single measurements, continuous monitoring of uACR can provide additional prognostic information. In patients with diabetic nephropathy, dynamic changes in uACR are closely related to renal outcomes. A large cohort study by T. Babazono et al. showed that patients with a ≥ 30% decrease in uACR within 1 year had significantly reduced risk of renal function deterioration (hazard ratio [HR] = 0.76), while a ≥ 30% increase in uACR significantly increased the risk of renal endpoint events (HR = 1.30) [15]. However, despite the important prognostic value of uACR in HFpEF patients, research on uACR trajectory patterns and their relationship with clinical outcomes in HFpEF patients remains very limited. Given the heterogeneity of HFpEF patients and the limitations of existing risk stratification tools, studying uACR trajectory patterns may provide new perspectives for identifying high‐risk HFpEF patients and optimizing management strategies. Therefore, we conducted this study analyzing data from the Treatment of Preserved Cardiac Function Heart Failure with an Aldosterone Antagonist (TOPCAT) trial to explore the relationship between different uACR trajectory patterns and clinical outcomes in HFpEF patients, aiming to provide new insights for risk stratification and personalized management of HFpEF.

## 2 Methods

### 2.1 Participants and Study Design

This study utilized data from the TOPCAT trial, a multicenter, international, randomized, double‐blind, placebo‐controlled study. TOPCAT was designed to evaluate the efficacy of spironolactone in reducing cardiovascular morbidity and mortality in patients with HFpEF. The trial enrolled 3445 patients aged 50 years or older with symptomatic heart failure and a left ventricular ejection fraction of 45% or higher from 270 sites across 6 countries between August 2006 and January 2012. Detailed information about the design, inclusion and exclusion criteria, and primary results of the TOPCAT trial have been previously published [16]. The study protocol complied with the Declaration of Helsinki and was approved by the institutional review board at each participating site. All participants provided written informed consent.

From the original TOPCAT cohort, we excluded: (1) patients for whom no urine data record was available in the trial database (*n* = 25), (2) patients without uACR measurements (*n* = 1230), (3) patients with invalid uACR values (*n* = 585), (4) patients with fewer than three uACR measurements during the follow‐up period (*n* = 858), and (5) patients with missing data on age or sex (*n* = 1). After applying these criteria, a total of 746 patients with HFpEF were included in the final analytical cohort. This was a post hoc secondary analysis; the hypotheses examined were not pre‐specified in the original trial protocol.

### 2.2 uACR Measurement and Trajectory Modeling

Urinary albumin and creatinine levels were measured at baseline and during follow‐up visits according to standard laboratory procedures. The uACR was calculated as urinary albumin (in milligrams) divided by urinary creatinine (in grams). Given the right‐skewed distribution of uACR values, a natural logarithmic transformation was applied prior to analysis. We identified distinctive patterns of uACR change over time using the Latent Class Trajectory Model with the lcmm package in R (Version 4.2.0). The model incorporated ln‐uACR as the dependent variable, with time as the independent variable, and adjusted for age and sex. Models with one to five latent classes were fitted. Model selection was based on the Bayesian information criterion (BIC), entropy, the requirement that each class comprises at least 5% of the total sample, and clinical interpretability. Four‐ and five‐class solutions yielded classes containing 0.0% and 0.9% of participants respectively and were rejected. Although the BIC favored the two‐class solution, the three‐class solution was retained on the basis of clinical interpretability, and the two‐class solution was examined as a sensitivity analysis. Fit statistics for all models are presented in Supporting Information S1: Table S1.

### 2.3 Clinical Outcomes and Follow‐up

The primary outcome was a composite endpoint consisting of death from cardiovascular causes, aborted cardiac arrest, or hospitalization for the management of heart failure. Secondary outcomes included all‐cause death, any hospitalization, heart failure hospitalization, aborted cardiac arrest, myocardial infarction, and stroke. All clinical events were adjudicated by the clinical endpoints committee according to the predefined criteria from the TOPCAT trial. Patients were evaluated every 4 months during the first year and every 6 months thereafter during the follow‐up period. The specific adjudication criteria for each outcome were referenced from the original TOPCAT study protocol.

### 2.4 Data Collection

The following participant information was collected from the TOPCAT database: Demographic data included age, sex, and race. Clinical measurements comprised body mass index (BMI), heart rate, systolic blood pressure (SBP), diastolic blood pressure (DBP), New York Heart Association (NYHA) class, and ejection fraction. Laboratory parameters were obtained from blood samples and consisted of complete blood count parameters (white blood cell [WBC], hemoglobin, platelets), liver function tests (alanine aminotransferase [ALT], aspartate aminotransferase [AST], alkaline phosphatase [ALP], albumin [Alb]), renal function markers (estimated glomerular filtration rate [eGFR], creatinine, blood urea nitrogen [BUN]), and metabolic indicators (glucose). Medical history information included chronic obstructive pulmonary disease (COPD), asthma, hypertension, diabetes mellitus, peripheral artery disease (PAD), hypercholesterolemia, atrial fibrillation, thyroid disease, and previous cardiovascular procedures (coronary artery bypass grafting [CABG], percutaneous coronary intervention [PCI], implantable cardioverter‐defibrillator [ICD], pacemaker). Medication use was also documented, including angiotensin‐converting enzyme inhibitors/angiotensin receptor blockers (ACEi/ARB), spironolactone, aspirin, beta blockers, diuretics, and statins.

### 2.5 Statistical Analysis

Baseline characteristics were compared across the three uACR trajectory groups. Continuous variables were first tested for normality using the Shapiro‐Wilk test. Normally distributed variables were presented as mean ± standard deviation and compared using one‐way ANOVA, while non‐normally distributed variables were presented as median (interquartile range) and compared using the Kruskal–Wallis test. Categorical variables were expressed as numbers and percentages and compared using chi‐square tests. The incidence of clinical outcomes among the three uACR trajectory groups was analyzed and compared using *χ* ^2^ tests. The association between uACR trajectory patterns and clinical outcomes was evaluated using Cox proportional hazards models. Three models with progressive adjustment were constructed: Model 1 (unadjusted); Model 2 (adjusted for age, sex, and race); and Model 3 (fully adjusted for age, sex, race, BMI, diastolic blood pressure, eGFR, hemoglobin, NYHA class, diabetes mellitus, COPD, hypercholesterolemia, atrial fibrillation, prior CABG, aspirin use, and statin use). Covariates of Model 3 were selected based on univariate Cox regression analyses with *p* < 0.05 for the composite endpoint. Kaplan–Meier curves were constructed to visualize the cumulative incidence of the composite endpoint according to uACR trajectory patterns, with differences assessed by the log‐rank test. To evaluate the incremental predictive value of uACR trajectory groups, we developed two Cox proportional hazards models for the composite endpoint. The base model included covariates from Model 3, while the full model additionally incorporated the uACR trajectory groups. Analyses of secondary endpoints were not adjusted for multiple comparisons and are therefore considered exploratory. We compared the discriminative ability of both models using Harrell's C‐index and time‐dependent receiver operating characteristic (ROC) curves at the median follow‐up (4 years) and the end of study (6 years). The likelihood ratio test was used to assess the statistical significance of the improvement after adding uACR trajectory groups. The relationship between clinical characteristics and membership in the uACR trajectory groups was examined using multinomial logistic regression, with the stable group serving as the reference category. Results were reported as odds ratios with 95% confidence intervals. Statistical significance was defined as *p* < 0.05, and all analyses were performed using R software (version 4.2.0).

Sensitivity analyses were conducted as follows. All covariates in the primary models were measured at baseline. To assess the influence of changes occurring during follow‐up, we constructed a counting‐process dataset in which follow‐up for each participant was divided into intervals defined by study visits and laboratory measurements (median 14 per participant; 10,726 in total), with each covariate taking the most recent value observed before the start of the interval. Extended Cox models were then fitted, with robust variance estimation clustered on participant. Covariates were handled in three ways. (1) Replaced: baseline diastolic blood pressure, eGFR and statin use from Model 3 were replaced by their time‐updated counterparts to avoid entering the same variable twice, with eGFR re‐calculated from serial serum creatinine using the CKD‐EPI equation (median 15 measurements per participant). (2) Retained: the remaining 12 Model 3 covariates (age, sex, race, body mass index, NYHA class, hemoglobin, chronic obstructive pulmonary disease, hypercholesterolemia, atrial fibrillation, prior coronary artery bypass grafting, aspirin use and diabetes mellitus) were kept as baseline values. (3) Added: variables not included in Model 3, comprising randomized treatment assignment (spironolactone vs. placebo, fixed throughout follow‐up) and the following time‐updated variables—systolic blood pressure; an indicator of ≥ 30% decline in eGFR from each participant's own baseline; use of angiotensin‐converting enzyme inhibitors or angiotensin receptor blockers, beta‐blockers, diuretics and glucose‐lowering agents; current study‐drug status and daily dose; and serum potassium. The proportional‐hazards assumption was assessed using scaled Schoenfeld residuals, and the interaction between uACR trajectory and randomized treatment was tested by likelihood ratio test.

## 3 Results

### 3.1 Baseline Characteristics

**Figure 1:**
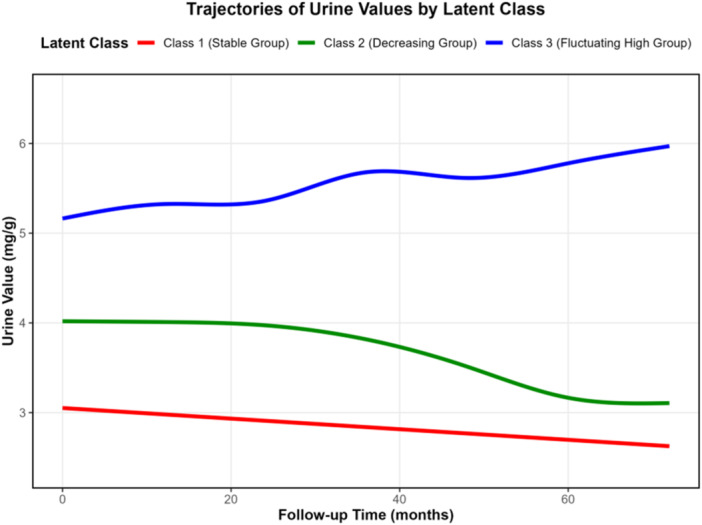
Identification of distinct urinary albumin/creatinine ratio (uACR) trajectory patterns in patients with HFpEF.

A total of 746 patients with HFpEF (mean age 70.58 ± 9.38 years, 54.56% male) were ultimately included in this study (Table 1). Three distinct uACR trajectory patterns were identified: stable group (*n* = 526, 70.5%), decreasing group (*n* = 123, 16.5%), and fluctuating high group (*n* = 97, 13.0%), as shown in Figure 1.

**Table 1:** Baseline characteristics of patients with HFpEF according to urinary albumin/creatinine ratio (uACR) trajectory patterns.

Variables	Total (*n* = 746)	Stable group (*n* = 526)	Decreasing group (*n *= 123)	Fluctuating high group (*n* = 97)	*p*
Age (years), mean ± SD	70.58 ± 9.38	70.17 ± 9.33	72.73 ± 9.38	70.01 ± 9.41	0.020
Sex, *n* (%)					< 0.001
Male	407 (54.56)	260 (49.43)	91 (73.98)	56 (57.73)	
Female	339 (45.44)	266 (50.57)	32 (26.02)	41 (42.27)	
Race, *n* (%)					0.061
White	654 (87.67)	461 (87.64)	114 (92.68)	79 (81.44)	
Black	69 (9.25)	46 (8.75)	7 (5.69)	16 (16.49)	
Other	23 (3.08)	19 (3.61)	2 (1.63)	2 (2.06)	
BMI (kg/m^2^), mean ± SD	32.43 ± 6.77	32.21 ± 6.79	32.35 ± 6.58	33.74 ± 6.81	0.124
Heart rate (bpm), mean ± SD	67.92 ± 10.39	68.25 ± 10.83	66.37 ± 9.75	68.11 ± 8.50	0.191
SBP (mmHg), mean ± SD	128.41 ± 13.58	128.57 ± 13.29	125.73 ± 14.36	130.93 ± 13.71	0.016
DBP (mmHg), mean ± SD	74.04 ± 10.62	75.13 ± 10.33	71.13 ± 10.75	71.81 ± 11.07	< 0.001
NYHA class, *n* (%)					0.127
I–II	537 (71.98)	390 (74.14)	82 (66.67)	65 (67.01)	
III–IV	209 (28.02)	136 (25.86)	41 (33.33)	32 (32.99)	
Ejection fraction (%), mean ± SD	57.16 ± 7.07	57.39 ± 7.08	56.51 ± 7.09	56.73 ± 6.99	0.375
eGFR (ml/min/1.73 m^2^), mean ± SD	65.04 ± 19.34	66.68 ± 19.02	61.70 ± 19.28	60.36 ± 20.00	0.001
Glucose (mmol/L), mean ± SD	6.60 ± 2.94	6.40 ± 2.61	6.71 ± 2.61	7.59 ± 4.46	0.001
Creatinine (μmol/L), mean ± SD	100.99 ± 26.75	96.78 ± 23.49	110.16 ± 29.06	112.20 ± 33.65	< 0.001
BUN (mg/dl), mean ± SD	22.02 ± 9.96	20.52 ± 8.73	24.33 ± 11.06	27.42 ± 12.32	< 0.001
WBC (×10^9^/L), mean ± SD	7.19 ± 2.34	7.07 ± 2.00	7.12 ± 1.61	7.95 ± 4.11	0.003
Hemoglobin (g/dl), mean ± SD	13.01 ± 1.57	13.06 ± 1.55	13.08 ± 1.62	12.69 ± 1.59	0.088
Platelets (×10^9^/L), mean ± SD	223.84 ± 67.33	226.52 ± 67.73	210.75 ± 68.07	225.85 ± 62.80	0.061
ALT (U/L), M (Q_1_, Q_3_)	22.00 (16.00, 32.00)	22.00 (16.00, 31.75)	22.00 (16.00, 31.50)	22.00 (16.00, 35.00)	0.586
Alp (U/L), M (Q_1_, Q_3_)	84.00 (66.00, 116.00)	86.00 (67.00, 117.00)	82.00 (62.00, 104.00)	86.00 (69.00, 118.00)	0.138
Ast (U/L), M (Q_1_, Q_3_)	22.40 (18.00, 27.95)	23.00 (18.00, 27.00)	22.00 (17.50, 28.00)	23.00 (18.00, 29.00)	0.555
Alb (g/dL), M (Q_1_, Q_3_)	4.00 (3.70, 4.30)	4.02 (3.70, 4.30)	3.91 (3.60, 4.20)	3.83 (3.60, 4.23)	0.023
COPD, *n* (%)	76 (10.19)	48 (9.13)	17 (13.82)	11 (11.34)	0.277
Asthma, *n* (%)	48 (6.43)	35 (6.65)	9 (7.32)	4 (4.12)	0.588
Hypertension, *n* (%)	672 (90.08)	472 (89.73)	111 (90.24)	89 (91.75)	0.828
Diabetes mellitus, *n* (%)	276 (37.00)	167 (31.75)	52 (42.28)	57 (58.76)	< 0.001
PAD, *n* (%)	67 (8.98)	48 (9.13)	8 (6.50)	11 (11.34)	0.450
Hypercholesterolemia, *n* (%)	494 (66.22)	321 (61.03)	95 (77.24)	78 (80.41)	< 0.001
Atrial Fibrillation, *n* (%)	337 (45.17)	236 (44.87)	59 (47.97)	42 (43.30)	0.761
Thyroid Disease, *n* (%)	115 (15.42)	84 (15.97)	12 (9.76)	19 (19.59)	0.109
CABG, *n* (%)	110 (14.75)	63 (11.98)	27 (21.95)	20 (20.62)	0.004
PCI, *n* (%)	125 (16.76)	70 (13.31)	27 (21.95)	28 (28.87)	< 0.001
ICD, *n* (%)	11 (1.47)	6 (1.14)	4 (3.25)	1 (1.03)	0.192
Pacemaker, *n* (%)	71 (9.52)	42 (7.98)	19 (15.45)	10 (10.31)	0.038
ACEi/ARB, *n* (%)	636 (85.25)	454 (86.31)	98 (79.67)	84 (86.60)	0.161
Spironolactone, *n* (%)	395 (52.95)	284 (53.99)	66 (53.66)	45 (46.39)	0.381
Aspirin, *n* (%)	499 (66.89)	345 (65.59)	82 (66.67)	72 (74.23)	0.251
Beta Blocker, *n* (%)	635 (85.12)	443 (84.22)	105 (85.37)	87 (89.69)	0.379
Diuretic, *n* (%)	701 (93.97)	496 (94.30)	112 (91.06)	93 (95.88)	0.278
Statin, *n* (%)	500 (67.02)	325 (61.79)	96 (78.05)	79 (81.44)	< 0.001

Abbreviations: ACEi, angiotensin‐converting enzyme inhibitor; ALT, alanine aminotransferase; ALP, alkaline phosphatase; ARB, angiotensin receptor blocker; AST, aspartate aminotransferase; BMI, body mass index; bpm, beats per minute; BUN, blood urea nitrogen; CABG, coronary artery bypass grafting; COPD, chronic obstructive pulmonary disease; DBP, diastolic blood pressure; eGFR, estimated glomerular filtration rate; HF, heart failure; ICD, implantable cardioverter‐defibrillator; NYHA, New York Heart Association; PAD, peripheral artery disease; PCI, percutaneous coronary intervention; SBP, systolic blood pressure; WBC, white blood cell.

Patients in the decreasing group were older and more likely to be male compared to the stable and fluctuating high groups (*p* < 0.05). The fluctuating high group had higher systolic blood pressure, while both decreasing and fluctuating high groups had lower diastolic blood pressure compared to the stable group (*p* < 0.05). Laboratory parameters showed that the fluctuating high group had higher glucose levels and WBC counts, but lower albumin levels (*p* < 0.05). Both decreasing and fluctuating high groups demonstrated worse renal function, with lower eGFR and higher creatinine and BUN levels (*p* < 0.05). Regarding comorbidities, diabetes mellitus was more prevalent in the fluctuating high group, while hypercholesterolemia was more common in both the fluctuating high and decreasing groups compared to the stable group (*p* < 0.001). History of cardiovascular procedures, including CABG and PCI, was more frequent in the decreasing and fluctuating high groups (*p* < 0.05). Correspondingly, statin use was also more prevalent in these two groups (*p* < 0.001). No significant differences were observed for other variables among the groups (all *p* > 0.05).

In a two‐class sensitivity analysis, 90 of the 97 participants assigned to the fluctuating high trajectory (92.8%) were classified into the higher‐risk class, which showed a comparable association with the primary endpoint (HR 1.72, 95% CI 1.15–2.59, *p* = 0.009).

### 3.2 Clinical Outcomes According to uACR Trajectory Patterns

**Figure 2:**
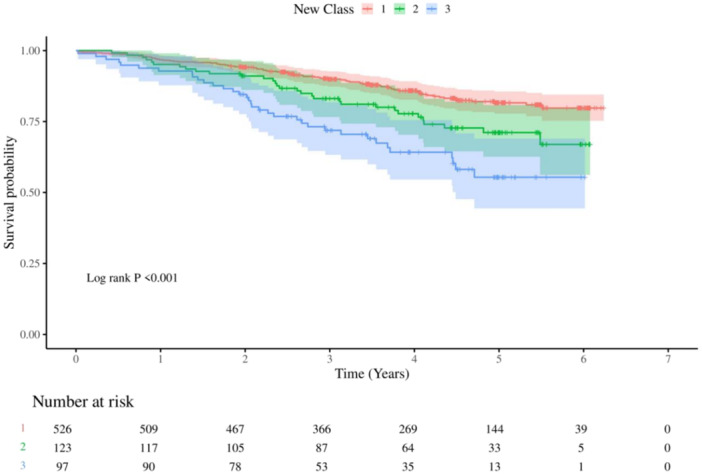
Kaplan–Meier curves for the composite endpoint according to urinary albumin/creatinine ratio (uACR) trajectory patterns in patients with HfpEF.

Table 2 presents the incidence of clinical outcomes stratified by uACR trajectory patterns. During follow‐up, 145 patients (19.44%) experienced the primary composite endpoint, with significantly higher incidence in the fluctuating high group (36.08%) compared to the decreasing (25.20%) and stable groups (15.02%) (*p* < 0.001). Kaplan–Meier analysis further confirmed significantly worse event‐free survival for the composite endpoint in both the fluctuating high and decreasing groups compared to the stable group (log‐rank *p* < 0.001, Figure 2).

**Table 2:** Incidence of clinical outcomes by uACR trajectory groups.

Endpoint	Total (*n* = 746)	Stable group (*n* = 526)	Decreasing group (*n* = 123)	Fluctuating high group (*n* = 97)	*p*
Composite endpoint, *n* (%)	145 (19.44)	79 (15.02)	31 (25.20)	35 (36.08)	< 0.001
Death, *n* (%)	65 (8.71)	38 (7.22)	15 (12.20)	12 (12.37)	0.083
Any hospitalization, *n* (%)	367 (49.20)	229 (43.54)	72 (58.54)	66 (68.04)	< 0.001
HF hospitalization, *n* (%)	114 (15.28)	62 (11.79)	23 (18.70)	29 (29.90)	< 0.001
Aborted cardiac arrest, *n* (%)	4 (0.54)	3 (0.57)	1 (0.81)	0 (0.00)	0.754
Myocardial infarction, *n* (%)	32 (4.29)	16 (3.04)	11 (8.94)	5 (5.15)	0.013
Stroke, *n* (%)	22 (2.95)	15 (2.85)	3 (2.44)	4 (4.12)	0.742

For secondary endpoints, significant differences were observed in any hospitalization (68.04% in fluctuating high, 58.54% in decreasing, and 43.54% in stable groups, *p* < 0.001), heart failure hospitalization (29.90% in fluctuating high, 18.70% in decreasing, and 11.79% in stable groups, *p* < 0.001), and myocardial infarction (8.94% in decreasing, 5.15% in fluctuating high, and 3.04% in stable groups, *p* = 0.013). No significant differences were observed for all‐cause death (*p* = 0.083), aborted cardiac arrest (*p* = 0.754) or stroke (*p* = 0.742).

### 3.3 Association Between uACR Trajectory Patterns and Clinical Outcomes

Table 3 presents the results of Cox proportional hazards analyses examining the association between uACR trajectory patterns and clinical outcomes across three increasingly adjusted models. For the primary composite endpoint, the unadjusted analysis (Model 1) showed that both the decreasing group (HR 1.69, 95% CI 1.12–2.57, *p* = 0.013) and fluctuating high group (HR 2.89, 95% CI 1.94–4.31, *p* < 0.001) were associated with significantly higher risk compared to the stable group. After adjusting for age, sex, and race (Model 2), the association remained significant for the fluctuating high group (HR 2.65, 95% CI 1.76–3.98, *p* < 0.001). In the fully adjusted model (Model 3), the fluctuating high group maintained a significant association with increased risk (HR 1.91, 95% CI 1.25–2.91, *p* = 0.003), while the decreasing group showed no significant association (HR 1.15, 95% CI 0.74–1.78, *p* = 0.536).

**Table 3:** Association between uACR trajectory patterns and clinical outcomes in patients with HFpEF.

Variables	Model1		Model2		Model3	
	HR (95%CI)	*p*	HR (95%CI)	*p*	HR (95%CI)	*p*
Composite endpoint						
Trajectories						
Stable group	1.00 (Reference)		1.00 (Reference)		1.00 (Reference)	
Decreasing group	1.69 (1.12 ~ 2.57)	0.013	1.51 (0.98 ~ 2.32)	0.061	1.15 (0.74 ~ 1.78)	0.536
Fluctuating high group	2.89 (1.94 ~ 4.31)	< 0.001	2.65 (1.76 ~ 3.98)	< 0.001	1.91 (1.25 ~ 2.91)	0.003
Death						
Trajectories						
Stable group	1.00 (Reference)		1.00 (Reference)		1.00 (Reference)	
Decreasing group	1.60 (0.88 ~ 2.91)	0.124	1.21 (0.65 ~ 2.24)	0.541	0.93 (0.49 ~ 1.75)	0.812
Fluctuating high group	2.01 (1.05 ~ 3.86)	0.035	1.78 (0.91 ~ 3.45)	0.090	1.23 (0.61 ~ 2.47)	0.566
Any hospitalization						
Trajectories						
Stable group	1.00 (Reference)		1.00 (Reference)		1.00 (Reference)	
Decreasing group	1.51 (1.16 ~ 1.97)	0.002	1.51 (1.15 ~ 1.99)	0.003	1.29 (0.97 ~ 1.71)	0.077
Fluctuating high group	2.00 (1.52 ~ 2.63)	< 0.001	1.84 (1.39 ~ 2.44)	< 0.001	1.49 (1.11 ~ 1.99)	0.008
Hosp for heart failure						
Trajectories						
Stable group	1.00 (Reference)		1.00 (Reference)		1.00 (Reference)	
Decreasing group	1.59 (0.98 ~ 2.56)	0.058	1.46 (0.89 ~ 2.40)	0.132	1.11 (0.67 ~ 1.83)	0.694
Fluctuating high group	2.94 (1.89 ~ 4.58)	< 0.001	2.74 (1.75 ~ 4.31)	< 0.001	1.91 (1.19 ~ 3.06)	0.007
Aborted cardiac arrest						
Trajectories						
Stable group	1.00 (Reference)		1.00 (Reference)		1.00 (Reference)	
Decreasing group	1.37 (0.14 ~ 13.22)	0.783	1.58 (0.16 ~ 15.76)	0.695	Inf (0.00 ~ Inf)	0.986
Fluctuating high group	0.00 (0.00 ~ Inf)	0.999	0.00 (0.00 ~ Inf)	0.999	0.00 (0.00 ~ Inf)	0.987
Adjudicated myocardial infarction						
Trajectories						
Stable group	1.00 (Reference)		1.00 (Reference)		1.00 (Reference)	
Decreasing group	2.97 (1.38 ~ 6.41)	0.005	3.18 (1.43 ~ 7.06)	0.005	3.00 (1.33 ~ 6.76)	0.008
Fluctuating high group	1.78 (0.65 ~ 4.87)	0.259	1.77 (0.64 ~ 4.87)	0.273	1.49 (0.52 ~ 4.25)	0.456
Stroke						
Trajectories						
Stable group	1.00 (Reference)		1.00 (Reference)		1.00 (Reference)	
Decreasing group	0.84 (0.24 ~ 2.90)	0.782	0.76 (0.21 ~ 2.74)	0.679	0.65 (0.18 ~ 2.36)	0.514
Fluctuating high group	1.57 (0.52 ~ 4.73)	0.425	1.64 (0.54 ~ 4.95)	0.381	1.43 (0.45 ~ 4.49)	0.544

*Note:* Model 1: Crude. Model 2: Adjusted for Age, Sex, Race Category. Model 3: Adjusted for Age, Sex, Race Category, BMI, DBP, eGFR, Hemoglobin, NYHA Class, Diabetes Mellitus, COPD, Hypercholesterolemia, Atrial Fibrillation, CABG, Aspirin, Statin.

Abbreviations: BMI, body mass index; CABG, coronary artery bypass grafting; CI, confidence interval; COPD, chronic obstructive pulmonary disease; DBP, diastolic blood pressure; eGFR, estimated glomerular filtration rate; HR, hazard ratio; NYHA, New York Heart Association.

For secondary endpoints, the fully adjusted model revealed that the fluctuating high group was significantly associated with increased risk of any hospitalization (HR 1.49, 95% CI 1.11–1.99, *p* = 0.008) and heart failure hospitalization (HR 1.91, 95% CI 1.19–3.06, *p* = 0.007) compared to the stable group. Notably, the decreasing group showed a higher risk of myocardial infarction (11 of 32 total events; HR 3.00, 95% CI 1.33–6.76, *p* = 0.008) after full adjustment. No significant associations were observed between uACR trajectory patterns and all‐cause death, aborted cardiac arrest, or stroke in the fully adjusted models.

### 3.4 Association Between Clinical Characteristics and uACR Trajectory Patterns

Table 4 demonstrates the associations between clinical characteristics and uACR trajectory patterns. Female sex was inversely associated with membership in the decreasing group (OR 0.31, 95% CI 0.20–0.50, *p* < 0.05), while showing no significant relationship with the fluctuating high group. Advanced age correlated with higher likelihood of belonging to the decreasing group (OR 1.04, 95% CI 1.01–1.07, *p* < 0.05). Diabetes mellitus emerged as a strong predictor of the fluctuating high pattern (OR 2.46, 95% CI 1.50–4.03, *p* < 0.05) without significant influence on the decreasing pattern. Hypercholesterolemia consistently predicted both non‐stable patterns, with similar odds ratios for the decreasing (OR 1.86, 95% CI 1.14–3.05, *p* < 0.05) and fluctuating high groups (OR 1.86, 95% CI 1.06–3.27, *p* < 0.05). Other examined clinical characteristics, including hypertension, BMI, NYHA class III–IV, and spironolactone use, demonstrated no significant associations with uACR trajectory patterns.

**Table 4:** Association between clinical characteristics and membership in urinary albumin/creatinine ratio (uACR) trajectory patterns in patients with HFpEF.

Potential_determinant	OR_CI
Sex, female	
Stable group	Reference
Decreasing group	0.31 (0.20–0.50)*
Fluctuating high group	0.77 (0.48–1.22)
Sex, male	
Stable group	Reference
Decreasing group	3.20 (2.02–5.06)*
Fluctuating high group	1.30 (0.82–2.07)
Hypertension	
Stable group	Reference
Decreasing group	1.00 (0.50–2.00)
Fluctuating high group	0.95 (0.43–2.14)
Diabetes mellitus	
Stable group	Reference
Decreasing group	1.30 (0.83–2.06)
Fluctuating high group	2.46 (1.50–4.03)*
Hypercholesterolemia	
Stable group	Reference
Decreasing group	1.86 (1.14–3.05)*
Fluctuating high group	1.86 (1.06–3.27)*
Age	
Stable group	Reference
Decreasing group	1.04 (1.01–1.07)*
Fluctuating high group	1.01 (0.98–1.03)
BMI	
Stable group	Reference
Decreasing group	1.00 (0.97–1.04)
Fluctuating high group	1.01 (0.97–1.04)
NYHA class, III–IV	
Stable group	Reference
Decreasing group	1.39 (0.88–2.20)
Fluctuating high group	1.19 (0.72–1.95)
Spironolactone	
Stable group	Reference
Decreasing group	0.95 (0.63–1.42)
Fluctuating high group	0.74 (0.47–1.16)

Abbreviations: BMI, body mass index; NYHA, New York Heart Association; OR_CI, odds ratio with 95% confidence interval.

* *p* < 0.05.

### 3.5 Predictive Value of uACR Trajectory Patterns

**Figure 3:**
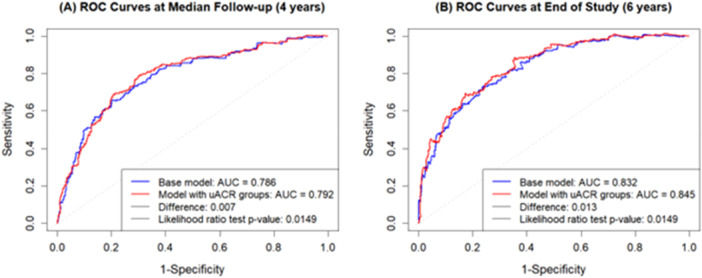
ROC curves comparing the predictive performance of models with and without uACR trajectory groups for composite endpoint at different follow‐up time points.

Figure 3 shows the time‐dependent ROC curves comparing models with and without uACR trajectory groups at different follow‐up time points. Adding uACR trajectory groups to the base model modestly improved risk prediction for the composite endpoint (likelihood ratio test *p* = 0.0149), with the C‐index increasing from 0.758 to 0.765. Time‐dependent ROC analysis showed that the AUC increased from 0.786 to 0.792 at median follow‐up (4 years; difference 0.007) and from 0.832 to 0.845 at study end (6 years; difference 0.013).

### 3.6 Sensitivity Analyses Accounting for Time‐Varying Confounding

Medication regimens changed frequently during follow‐up: 630 participants (84.4%) had a change in diuretic use, and 588 (78.8%) in angiotensin‐converting enzyme inhibitor or angiotensin receptor blocker use. After additional adjustment for randomized treatment assignment (spironolactone vs placebo), the association between the fluctuating high trajectory and the primary endpoint was unchanged (HR 1.88, 95% CI 1.23–2.88, *p* = 0.004), and it persisted in the fully adjusted model additionally incorporating time‐updated blood pressure, eGFR, concomitant medication use and study‐drug exposure (HR 1.90, 95% CI 1.22–2.95, *p* = 0.004). The decreasing trajectory was not significantly associated with the primary endpoint in any model (fully adjusted HR 1.26, 95% CI 0.81–1.95, *p* = 0.302). Details are provided in Supporting Information S1: Tables S2 and S3.

## 4 Discussion

In this study, we identified three distinct uACR trajectory patterns among 746 HFpEF patients from the TOPCAT trial using latent class mixed‐effects modeling: stable group (70.5%), decreasing group (16.5%), and fluctuating high group (13.0%), and explored their associations with clinical outcomes. Our analysis revealed several important findings. First, the fluctuating high group was significantly associated with increased risk of the primary composite endpoint (HR 1.91, 95% CI 1.25–2.91), even after adjusting for multiple known risk factors. Second, different trajectory groups showed differential associations with specific clinical outcomes: the fluctuating high group primarily predicted heart failure hospitalization (HR 1.91, 95% CI 1.19–3.06), while the decreasing group specifically predicted myocardial infarction risk (HR 3.00, 95% CI 1.33–6.76). Third, we found specific demographic characteristics and clinical factors closely associated with different trajectory patterns, such as male sex strongly correlating with the decreasing trajectory (OR 3.20), and diabetes strongly correlating with the fluctuating high trajectory (OR 2.46), suggesting gender differences and metabolic status may be important factors influencing uACR dynamic changes. Finally, incorporating uACR trajectory information into prediction models significantly improved risk prediction capacity, particularly during long‐term follow‐up (AUC increased from 0.832 to 0.845 at 6 years). These results provide a new dimension for risk assessment in HFpEF patients, potentially helping to identify high‐risk patients and optimize individualized treatment strategies.

In our study, the fluctuating high group exhibited the highest risk for the composite endpoint (HR 1.91) and heart failure hospitalization (HR 1.91), which may reflect specific pathophysiological mechanisms. First, fluctuating high albuminuria may represent a more severe endothelial dysfunction phenotype characterized by instability in endothelial injury‐repair processes rather than a steady state of damage [6]. As vascular endothelium struggles to complete adequate repair under recurrent stress, this cyclic variation may cause greater cumulative damage to the vascular system, thereby increasing cardiovascular event risk. Second, metabolic abnormalities, particularly diabetes, likely play a central role in this trajectory formation. Our study demonstrated that diabetes was not only a strong predictor of the fluctuating high trajectory (OR 2.46) but also significantly more prevalent in this group compared to the stable group (58.76% vs. 31.75%, *p* < 0.001). Previous research has confirmed that blood glucose fluctuations directly affect endothelial function and glomerular filtration membrane permeability [8], potentially explaining why diabetic patients are more likely to exhibit fluctuating albuminuria. Additionally, the higher prevalence of hypercholesterolemia (80.41%) in this group may further compromise endothelial glycocalyx integrity through oxidative stress and lipid peroxidation [10], exacerbating this unstable state. Third, impaired renal function (lower eGFR and higher creatinine levels) in the fluctuating high group reflects the importance of cardiorenal interaction in HFpEF. Renal dysfunction leads to fluid retention and neurohormonal dysregulation, directly worsening heart failure symptoms [14, 17, 18]. In this state, intermittent glomerular filtration membrane damage may be the direct mechanism of albuminuria fluctuation, with persistent inflammation and oxidative stress further aggravating this damage. Collectively, the fluctuating high uACR trajectory may represent a distinct cardiorenal syndrome phenotype characterized by endothelial instability driven by metabolic disorders (especially diabetes), promoting renal function impairment and heart failure progression. Identifying this phenotype has important clinical implications for risk stratification and individualized treatment strategies in HFpEF patients, particularly for providing more precise prognostic assessment and management plans for high‐risk patients with diabetes and HFpEF.

An unexpected but important finding in our study was that while the decreasing group's risk prediction for the composite endpoint was no longer significant after comprehensive adjustment, its association with myocardial infarction risk persisted (HR 3.00, *p* = 0.008), a phenomenon worthy of in‐depth discussion. First, this group showed significant gender differences, with males significantly overrepresented compared to other groups (73.98% vs 49.43%, *p* < 0.001), consistent with male sex being a known risk factor for myocardial infarction. Our multinomial logistic regression analysis further confirmed that compared to females, males were 3.2 times more likely to belong to the decreasing group (OR 3.20). This gender difference may be related to the protective effect of estrogen on endothelial function, and the absence of such protection may lead to vascular vulnerability even when albuminuria improves. Second, the reduction in uACR in the decreasing group may reflect superficial improvement while masking underlying compensatory mechanisms. For example, compensatory activation of the renin‐angiotensin‐aldosterone system (RAAS) may lead to blood pressure regulation disorder, which might reduce proteinuria but simultaneously increase the risk of coronary events. This is consistent with the lower diastolic blood pressure (71.13 vs. 75.13 mmHg, *p* < 0.001) and higher CABG rate (21.95% vs. 11.98%, *p* = 0.004) in this group. Furthermore, the higher proportion of hypercholesterolemia (77.24% vs. 61.03%, *p* < 0.001) and corresponding increased statin use may indicate that while lipid‐lowering therapy improved albuminuria status, it failed to completely reverse existing vascular damage. Particularly noteworthy is that patients in this group were older (72.73 vs. 70.17 years, *p* = 0.020), and age was a predictor of this trajectory (OR 1.04), suggesting that the cumulative effect of long‐term vascular damage may be difficult to fully reverse through albuminuria reduction. Overall, this phenomenon suggests that in HFpEF patients, a decreasing uACR trajectory may represent a state of “pseudo‐improvement,” where the underlying vascular pathological process may continue despite reduced albuminuria, particularly regarding coronary event risk. These mechanisms are speculative and require confirmation in studies with a larger number of coronary events.

Our study offers several important innovations in assessing the relationship between uACR and HFpEF prognosis. First, we employed trajectory analysis rather than traditional single measurements, providing crucial information about dynamic changes. Katz et al. (2014) previously reported that a single elevated uACR was closely associated with left ventricular remodeling, right ventricular dysfunction, and adverse cardiovascular outcomes in HFpEF patients [19], and de Boer et al. (2018) also confirmed that elevated baseline uACR significantly increased HFpEF risk (HR 1.33, 95%CI 1.20–1.48) [20]. However, these studies failed to capture the dynamic changes in uACR. We not only confirmed the prognostic value of uACR but also revealed associations between specific trajectory patterns and specific clinical outcomes, such as the fluctuating high group with heart failure hospitalization and the decreasing group with myocardial infarction risk. More importantly, our time‐dependent ROC analysis showed that the improvement in predictive ability from uACR trajectory information increased with longer follow‐up, with a slightly larger increase in AUC at 6 years (from 0.832 to 0.845). This suggests that dynamic change patterns may reflect long‐term trends in disease progression. Second, our research complements other biomarker trajectory analyses beneficially. Liu et al. (2024) analyzed systolic blood pressure trajectories based on TOPCAT data and found that both “elevated” and “decreased” trajectory groups had higher cardiovascular event risk than the “stable” group [21]. Similarly, Xing et al. (2025) using TOPCAT data found that the “recurrent worsening” trajectory group for depressive symptoms had significantly increased all‐cause mortality risk (HR 2.05) [22]. Our study applied trajectory analysis to uACR in HFpEF patients for the first time, finding that the “fluctuating high” trajectory was associated with the worst prognosis, consistent with the conclusion from the above studies that “unstable” trajectories predict higher risk. Third, by comparing with uACR trajectory studies in diabetic patients, we identified disease‐specific patterns. Li et al. (2024) identified four uACR trajectories in diabetic patients, with both “high stable” and “elevated‐increasing” groups showing significantly increased risk of major cardiovascular events (HRs of 1.337 and 1.648, respectively) [23]. While we also observed high uACR associated with poor prognosis in HFpEF, we emphasized the unique value of the fluctuating pattern (HR 1.91). This difference may reflect different pathophysiological mechanisms between the two diseases: diabetes primarily affects prognosis through persistent microvascular damage, while HFpEF may be more influenced by hemodynamic fluctuations and intermittent renal function impairment, suggesting that uACR trajectory analysis can capture disease‐specific risk patterns even across different patient populations. In summary, our study expanded the understanding of the prognostic value of uACR in HFpEF through trajectory analysis, providing an empirical basis for refined risk stratification and individualized treatment decisions, while also laying the foundation for future comprehensive assessment models combining multiple biomarker trajectories.

Our study provides valuable insights into the relationship between uACR trajectory patterns and HFpEF patient outcomes, but several limitations should be considered. First, as a secondary analysis of the TOPCAT trial, which was originally designed to evaluate the efficacy of aldosterone antagonists in HFpEF patients rather than specifically studying uACR trajectory patterns, the timing intervals and frequency of uACR measurements were not optimized for trajectory analysis, potentially affecting the detection of certain short‐term fluctuation patterns. For the same reason, uACR was not measured in all participants, and trajectory modeling required at least three valid measurements, so only 746 of the 3445 randomized patients could be analyzed. The analytical cohort was therefore restricted to participants with more complete longitudinal data, and selection bias cannot be excluded. Second, although our analysis included 746 patients and identified three distinct trajectory patterns, some subgroups (such as the decreasing and fluctuating high groups) had relatively small sample sizes, which may have limited the statistical power for secondary endpoint analyses. Third, despite adjustment for multiple clinically relevant confounding factors, including time‐updated blood pressure, renal function, medication use, and study‐drug exposure, some variables remained unavailable: HbA1c and repeated fasting glucose were not collected during follow‐up in TOPCAT, so glycaemic control could be approximated only by use of glucose‐lowering therapy, and medication adherence and dietary habits were not recorded. In addition, post‐baseline blood pressure, renal function, and medication changes may lie on the causal pathway between uACR trajectory and outcome; these were therefore examined in sensitivity analyses, with the originally specified model retained as primary. Furthermore, our study provides evidence of associations between uACR trajectories and clinical outcomes, but cannot fully elucidate the underlying causal mechanisms, particularly the specific pathophysiological processes behind the fluctuating high trajectory. Finally, while the TOPCAT trial provided high‐quality data, its inclusion criteria and regional distribution characteristics may require further validation of our findings in broader HFpEF populations.

Based on our findings, future research could explore several directions: First, conducting prospective studies specifically focused on uACR dynamic changes, using more frequent and regular measurement protocols to validate and refine trajectory classification. Second, combining imaging and biomarkers to explore mechanisms underlying different uACR trajectory patterns, particularly the association between the fluctuating high pattern and endothelial dysfunction and inflammation. Third, evaluating individualized treatment strategies for different trajectory patterns, such as intensified diabetes management for the fluctuating high group, or enhanced coronary event prevention for the decreasing group. Additionally, given the special association between the decreasing group and myocardial infarction, in‐depth research on the role of gender differences in this phenomenon and the potential cardiovascular protective mechanisms of estrogen in HFpEF patients is also valuable. Finally, integrating uACR trajectory information with other known risk prediction factors to build more comprehensive risk assessment models has the potential to provide new tools for precise risk stratification in HFpEF patients and provide a more solid foundation for clinical decision‐making.

## 5 Conclusion

Our study identified three distinct uACR trajectory patterns in HFpEF patients, with the fluctuating high pattern independently associated with adverse cardiovascular outcomes (HR 1.91) and the decreasing pattern showed an exploratory association with myocardial infarction (HR 3.00) that requires validation. Importantly, trajectory patterns were strongly predicted by sex and diabetes, suggesting underlying pathophysiological mechanisms. By demonstrating a modest incremental predictive value of uACR trajectories, our findings provide a novel risk stratification approach for HFpEF patients, potentially enabling more targeted interventions and improved outcomes in this challenging patient population.

## Author Contributions

W.X., M.P., and X.Z. were in charge of methodology and wrote the manuscript. Z.W. and D.Y. analyzed the data and revised the manuscript. All authors approved the final manuscript.

## Funding

This study was funded by the Capital Medical University‐Digital intelligence basic clinical cooperation SZHL23M10.

## Ethics Statement

Informed consent for data use has been described in previous studies with the subject's own authorization. Additionally, the current research protocol has been approved by the ethics committee of Ji'an Central People's Hospital, and the entire research process follows the Declaration of Helsinki.

## Conflicts of Interest

The authors declare no conflicts of interest.

## Supporting information


Supporting File

## Data Availability

The data used in this study were obtained from the Treatment of Preserved Cardiac Function Heart Failure with an Aldosterone Antagonist (TOPCAT) trial, publicly available through the National Heart, Lung, and Blood Institute (NHLBI) Biologic Specimen and Data Repository Information Coordinating Center (BioLINCC) at https://biolincc.nhlbi.nih.gov/.

## References

[1] B. A. Borlaug , K. Sharma , S. J. Shah , and J. E. Ho , “Heart Failure With Preserved Ejection Fraction,” Journal of the American College of Cardiology 81 (2023): 1810–1834. 37137592 10.1016/j.jacc.2023.01.049

[2] F. Gentile , N. Ghionzoli , C. Borrelli , et al., “Epidemiological and Clinical Boundaries of Heart Failure With Preserved Ejection Fraction,” European Journal of Preventive Cardiology 29 (2022): 1233–1243. 33963839 10.1093/eurjpc/zwab077

[3] P. Li , H. Zhao , J. Zhang , et al., “Similarities and Differences Between Hfmref and hfpef,” Frontiers in Cardiovascular Medicine 8 (2021): 678614. 34616777 10.3389/fcvm.2021.678614 PMC8488158

[4] B. A. Borlaug , “Evaluation and Management of Heart Failure With Preserved Ejection Fraction,” Nature Reviews Cardiology 17 (2020): 559–573. 32231333 10.1038/s41569-020-0363-2

[5] R. Zakeri and M. R. Cowie , “Heart Failure With Preserved Ejection Fraction: Controversies, Challenges and Future Directions,” Heart 104 (2018): 377–384. 29305560 10.1136/heartjnl-2016-310790

[6] D. de Zeeuw , H. H. Parving , and R. H. Henning , “Microalbuminuria as An Early Marker for Cardiovascular Disease,” Journal of the American Society of Nephrology 17 (2006): 2100–2105. 16825327 10.1681/ASN.2006050517

[7] M. S. Khan , I. Shahid , S. D. Anker , et al., “Albuminuria and Heart Failure,” Journal of the American College of Cardiology 81 (2023): 270–282. 36653095 10.1016/j.jacc.2022.10.028

[8] C. R. Parikh and S. G. Coca , “Permissive Aki’ With Treatment of Heart Failure,” Kidney International 96 (2019): 1066–1068. 31648696 10.1016/j.kint.2019.07.003

[9] F. Xia , G. Liu , Y. Shi , and Y. Zhang , “Impact of Microalbuminuria on Incident Coronary Heart Disease, Cardiovascular and All‐Cause Mortality: A Meta‐Analysis of Prospective Studies,” International Journal of Clinical and Experimental Medicine 8 (2015): 1–9. 25784968 PMC4358423

[10] A. Jager , P. J. Kostense , H. G. Ruhé , et al., “Microalbuminuria and Peripheral Arterial Disease Are Independent Predictors of Cardiovascular and All‐Cause Mortality, Especially Among Hypertensive Subjects: Five‐Year Follow‐Up of the Hoorn Study,” Arteriosclerosis, Thrombosis, and Vascular Biology 19 (1999): 617–624. 10073965 10.1161/01.atv.19.3.617

[11] R. Donnelly , J. M. Yeung , and G. Manning , “Microalbuminuria: A Common, Independent Cardiovascular Risk Factor, Especially but Not Exclusively in Type 2 Diabetes,” Journal of hypertension. Supplement: official journal of the International Society of Hypertension 21 (2003): 7–12. 12769161

[12] J. S. Jensen , B. Feldt‐Rasmussen , S. Strandgaard , M. Schroll , and K. Borch‐Johnsen , “Arterial Hypertension, Microalbuminuria, and Risk of Ischemic Heart Disease,” Hypertension 35 (2000): 898–903. 10775558 10.1161/01.hyp.35.4.898

[13] S. Masson , R. Latini , V. Milani , et al., “Prevalence and Prognostic Value of Elevated Urinary Albumin Excretion in Patients With Chronic Heart Failure: Data From the Gissi‐Heart Failure Trial,” Circulation: Heart Failure 3 (2010): 65–72. 19850697 10.1161/CIRCHEARTFAILURE.109.881805

[14] C. E. Jackson , S. D. Solomon , H. C. Gerstein , et al., “Albuminuria in Chronic Heart Failure: Prevalence and Prognostic Importance,” Lancet 374 (2009): 543–550. 19683640 10.1016/S0140-6736(09)61378-7

[15] T. Babazono , K. Hanai , Y. Yokoyama , and K. Uchiyama , “Association Between 1‐Year Changes in Urinary Albumin‐To‐Creatinine Ratio and Kidney Disease Progression in Japanese Individuals With Diabetes: A Historical Cohort Study,” Clinical and Experimental Nephrology 27 (2023): 1001–1009. 37606804 10.1007/s10157-023-02380-8 PMC10654190

[16] B. Pitt , M. A. Pfeffer , S. F. Assmann , et al., “Spironolactone for Heart Failure With Preserved Ejection Fraction,” New England Journal of Medicine 370 (2014): 1383–1392. 24716680 10.1056/NEJMoa1313731

[17] L. Shan , K. Zheng , W. Dai , P. Hao , and Y. Wang , “J‐Shaped Association Between Serum Glucose Potassium Ratio and Prognosis in Heart Failure With Preserved Ejection Fraction With Stronger Predictive Value in Non‐Diabetic Patients,” Scientific Reports 14 (2024): 29965. 39622960 10.1038/s41598-024-81289-y PMC11612494

[18] D. Su , F. Wang , Y. Yang , et al., “The Association Between Frailty and In‐Hospital Mortality in Critically Ill Patients With Congestive Heart Failure: Results From Mimic‐Iv Database,” Frontiers in Cardiovascular Medicine 11 (2024): 1361542. 38863896 10.3389/fcvm.2024.1361542 PMC11165203

[19] D. H. Katz , J. A. Burns , F. G. Aguilar , L. Beussink , and S. J. Shah , “Albuminuria Is Independently Associated With Cardiac Remodeling, Abnormal Right and Left Ventricular Function, and Worse Outcomes in Heart Failure With Preserved Ejection Fraction,” JACC: Heart Failure 2 (2014): 586–596. 25282032 10.1016/j.jchf.2014.05.016 PMC4256131

[20] R. A. de Boer , M. Nayor , C. R. deFilippi , et al., “Association of Cardiovascular Biomarkers With Incident Heart Failure With Preserved and Reduced Ejection Fraction,” JAMA Cardiology 3 (2018): 215–224. 29322198 10.1001/jamacardio.2017.4987 PMC5862778

[21] X. Liu , H. Pan , Y. Jiang , et al., “Association Between Trajectory of Systolic Blood Pressure and Outcomes in Heart Failure Patients With Preserved Ejection Fraction (hfpef),” European Journal of Internal Medicine 131 (2025): 89–97. 39419733 10.1016/j.ejim.2024.09.003

[22] F. Xing , M. Gao , Y. Wu , et al., “Influence of Depression Trajectories in Heart Failure Patients With Preserved Ejection Fractions: A Secondary Analysis of Adverse Outcomes in the Topcat Trial,” Heart 111 (2025): 733–740. 40089312 10.1136/heartjnl-2024-324505

[23] H. Li , Y. Ren , Y. Duan , P. Li , and Y. Bian , “Association of the Longitudinal Trajectory of Urinary Albumin/Creatinine Ratio in Diabetic Patients With Adverse Cardiac Event Risk: A Retrospective Cohort Study,” Frontiers in Endocrinology 15 (2024): 1355149. 38745945 10.3389/fendo.2024.1355149 PMC11091466

