# Supplementary material for: Dynamic Urinary Albumin/Creatinine Ratio Patterns Predict Adverse Outcomes in HFpEF: TOPCAT Cohort Analysis
Source: Clin Cardiol. 2026 Aug 14;49(8):e70446. doi: 10.1002/clc.70446 (PMC13475011; doi:10.1002/clc.70446)
Supplement: Supplementary file 1 [file CLC-49-e70446-s001.docx]

**Supplementary Material**

**Supplementary Table S1. Fit statistics for latent class trajectory models with one to five classes**

| **Number of classes** | **Log-likelihood** | **Parameters** | **BIC** | **Entropy** | **Smallest class (%) / mean posterior probability** |
| --- | --- | --- | --- | --- | --- |
| 1 | −5091.8 | 8 | 10236.6 | — | — |
| 2 | −5021.6 | 13 | 10129.2 | 0.769 | 12.7 / 0.895 |
| **3 (selected)** | **−5017.2** | **18** | **10153.4** | **0.401** | **13.0 / 0.669** |
| 4 | −5017.2 | 23 | 10186.5 | 0.323 | 0.0 / 0.606 |
| 5 | −4994.8 | 28 | 10174.8 | 0.525 | 0.9 / 0.648 |

All models converged. Model selection was based on the Bayesian information criterion, entropy, a minimum class size of 5% of the total sample, and clinical interpretability. Four- and five-class solutions produced classes containing 0.0% and 0.9% of participants respectively and were not considered further. BIC, Bayesian information criterion.

**Supplementary Table S2. Association between uACR trajectory and the primary endpoint under progressive adjustment for time-varying confounders**

| **Model** | **Decreasing HR (95% CI)** | **P** | **Fluctuating high HR (95% CI)** | **P** | **Events** |
| --- | --- | --- | --- | --- | --- |
| Model 3 (covariates fixed at baseline) | 1.15 (0.74–1.78) | 0.536 | 1.91 (1.25–2.91) | 0.003 | 145 |
| ＋ randomized treatment assignment | 1.15 (0.74–1.79) | 0.527 | 1.88 (1.23–2.88) | 0.004 | 145 |
| ＋ all time-varying covariates | 1.26 (0.81–1.95) | 0.302 | 1.90 (1.22–2.95) | 0.004 | 142 |

Reference category is the stable trajectory. Time-varying covariates are listed in Supplementary Table S3. Baseline diastolic blood pressure, eGFR and statin use from Model 3 were replaced by their time-updated counterparts; the remaining Model 3 covariates were retained at baseline. Three events were excluded from the fully adjusted model owing to missing covariate data. CI, confidence interval; HR, hazard ratio; uACR, urinary albumin-to-creatinine ratio.

**Supplementary Table S3. Covariate hazard ratios in the fully adjusted time-varying model**

| **Covariate** | **HR (95% CI)** | **P** |
| --- | --- | --- |
| *uACR trajectory (ref. stable)* |  |  |
| Decreasing | 1.26 (0.81–1.95) | 0.302 |
| Fluctuating high | 1.90 (1.22–2.95) | 0.004 |
| Systolic blood pressure, per 1 SD | 0.89 (0.74–1.07) | 0.206 |
| Diastolic blood pressure, per 1 mmHg | 1.01 (0.99–1.03) | 0.205 |
| eGFR, per 1 SD | 0.69 (0.54–0.87) | 0.002 |
| eGFR decline ≥30% from baseline | 1.12 (0.69–1.82) | 0.635 |
| ACE inhibitor / ARB use | 0.63 (0.39–0.99) | 0.046 |
| Beta-blocker use | 0.77 (0.48–1.24) | 0.289 |
| Diuretic use | 1.89 (1.00–3.58) | 0.051 |
| Statin use | 1.51 (0.84–2.70) | 0.169 |
| Glucose-lowering therapy | 0.92 (0.49–1.70) | 0.784 |
| Currently receiving study drug | 0.86 (0.42–1.77) | 0.680 |
| Study-drug daily dose, per 1 mg | 1.00 (0.98–1.02) | 0.763 |
| Serum potassium, per 1 SD | 1.03 (0.86–1.23) | 0.774 |
| Randomized to spironolactone | 0.73 (0.50–1.07) | 0.109 |

All covariates listed are time-updated except randomized treatment assignment. Baseline Model 3 covariates retained in the model (age, sex, race, body mass index, NYHA class, haemoglobin, chronic obstructive pulmonary disease, hypercholesterolaemia, atrial fibrillation, prior coronary artery bypass grafting, aspirin use and diabetes mellitus) are not shown. Analysis comprised 142 events among 746 participants across 10,726 intervals. ACE, angiotensin-converting enzyme; ARB, angiotensin receptor blocker; CI, confidence interval; eGFR, estimated glomerular filtration rate; HR, hazard ratio; SD, standard deviation.
